# Checklist of the family Ceratopogonidae (Diptera) of Finland

**DOI:** 10.3897/zookeys.441.7742

**Published:** 2014-09-19

**Authors:** Larry Huldén, Lena Huldén

**Affiliations:** 1 Finnish Museum of Natural History, Zoology Unit, P.O. Box 17, FI-00014 University of Helsinki, Finland; 2 University of Helsinki, Department of Agricultural Sciences, P.O. Box 27, FI-00014 University of Helsinki, Finland

**Keywords:** Checklist, Finland, Diptera, Ceratopogonidae

## Abstract

A checklist of the family Ceratopogonidae (Diptera) recorded from Finland is provided.

## Introduction

The biting midges (Ceratopogonidae) have only occasionally been studied by Finnish entomologists. Most notable are the studies by Lundström (1910, 1916), Munsterhjelm (1920), Storå (1939) and Hämäläinen (1988). The last mentioned reference is not published in print but included because the determinations have been checked by Peter Havelka. Other species reports were given by Frey (1918), Forsius (1924), Krogerus (1960), Meinander (1977) and Hildén et al. (2010). A considerable part of the species information has been contributed by foreign specialists, Edwards (1924), Clastrier (1961, 1991), Delecolle et al. (1983), Borkent and Bissett (1990), Szadziewski (2001), Ashe et al. (2007), Dominiak et al. (2007), Dominiak and Szadziewski (2010) and Dominiak (2012).

A World Catalogue of Ceratopogonidae is maintained by Art Borkent (2014), updated Jan. 20, 2014.

**Number of species:**

World: 6180 extant species + 274 fossil species (Borkent 2014)

Europe: 567 species (Fauna Europaea, Szadzievski, Borkent and Dominiak 2014)

Finland: 97 species

Faunistic knowledge level in Finland: medium

## Checklist

suborder Nematocera Dumeril, 1805

infraorder Culicomorpha Hennig, 1948

superfamily Chironomoidea Newman, 1834

**CERATOPOGONIDAE** Newman, 1834

FORCIPOMYIINAE Lenz, 1934

***ATRICHOPOGON*** Kieffer, 1906

**sg. *Atrichopogon*** Kieffer, 1906

*Atrichopogon brunnipes* (Meigen, 1804)

*Atrichopogon fuscus* (Meigen, 1804)

= *fossicola* (Kieffer, 1922)

*Atrichopogon longicalcar* Remm, 1961

*Atrichopogon minutus* (Meigen, 1830)

*Atrichopogon pavidus* (Winnertz, 1852)

**sg. *Meloehelea*** Wirth, 1956

*Atrichopogon lucorum* (Meigen, 1818)

*Atrichopogon meloesugans* Kieffer, 1922

= *winnertzi* auct. nec Goetghebuer, 1922

*Atrichopogon oedemerarum* Storå, 1939

**sg. *Psilokempia*** Enderlein, 1936

*Atrichopogon appendiculatus* (Goetghebuer, 1920)

*Atrichopogon forcipatus* (Winnertz, 1852)

*Atrichopogon maculatus* (Lundström, 1910)

= minutus var. maculatus Lundström, 1910

*Atrichopogon rostratus* (Winnertz, 1852)

***FORCIPOMYIA*** Meigen, 1818

**sg. *Caloforcipomyia*** Saunders, 1957

*Forcipomyia glauca* Macfie, 1934

**sg. *Forcipomyia*** Meigen, 1818

*Forcipomyia bipunctata* (Linnaeus, 1767)

*Forcipomyia ciliata* (Winnertz, 1852)

*Forcipomyia costata* (Zetterstedt, 1838)

= *picea* (Winnertz, 1852)

*Forcipomyia kaltenbachi* (Winnertz, 1852)

*Forcipomyia myrmecophila* (Egger, 1863)

*Forcipomyia nigra* (Winnertz, 1852)

**sg. *Microhelea*** Kieffer, 1917

*Forcipomyia fuliginosa* (Meigen, 1818)

= *brevimana* (Lundström, 1910)

**sg. *Thyridomyia*** Saunders, 1925

*Forcipomyia monilicornis* (Coquillett, 1905)

= *hirta* (Lundström, 1910)

**sg. *Trichohelea*** Goetghebuer, 1920

*Forcipomyia eques* (Johanssen, 1908)

DASYHELEINAE Lenz, 1934

***DASYHELEA*** Kieffer, 1911

**sg. *Dasyhelea*** Kieffer, 1911

*Dasyhelea bensoni* Edwards, 1933

= *vernalis* Remm, 1979

*Dasyhelea bilineata* Goetghebuer, 1920

= *saxicola* (Edwards, 1929)

= *obscura* auct. nec (Winnertz, 1852)

= *versicolor* auct. nec (Winnertz, 1852)

*Dasyhelea pallidiventris* (Goetghebuer, 1931)

**sg. *Dicryptoscena*** Enderlein, 1936

*Dasyhelea modesta* (Winnertz, 1852)

= *aestiva* (Winnertz, 1852)

= *longipalpis* Kieffer, 1913

*Dasyhelea notata* Goetghebuer, 1920

**sg. *Pseudoculicoides*** Malloch, 1915

*Dasyhelea corinneae* Gosseries, 1991

= *scutellata* (Meigen, 1830), preocc.

*Dasyhelea flavoscutellata* (Zetterstedt, 1850)

= *egens* (Winnertz, 1852)

**sg. *Sebessia*** Remm, 1979

*Dasyhelea holosericea* (Meigen, 1804)

CERATOPOGONINAE Newman 1834

tribe Culicoidini Kieffer, 1911

***Culicoides*** Latreille, 1809

*Culicoides achrayi* Kettle & Lawson, 1955

*Culicoides albicans* (Winnertz, 1852)

= *vexans* auct. nec (Staeger, 1839)

*Culicoides chiopterus* (Meigen, 1830)

*Culicoides circumscriptus* Kieffer, 1918

*Culicoides dewulfi* Goetghebuer, 1936

*Culicoides fascipennis* (Staeger 1839)

*Culicoides festivipennis* Kieffer, 1914

= *odiblis* Austen, 1921

*Culicoides grisescens* Edwards, 1939

*Culicoides impunctatus* Goetghebuer, 1920

*Culicoides kibunensis* Tokunaga 1937

= *cubitalis* Edwards in Edwards et al. 1939

*Culicoides nubeculosus* (Meigen, 1830)

*Culicoides obsoletus* (Meigen, 1818)

= *varius* (Winnertz, 1852)

*Culicoides pallidicornis* (Kieffer 1919)

*Culicoides pictipennis* (Staeger, 1839)

*Culicoides pseudoheliophilus* Callot & Kremer, 1961

*Culicoides pulicaris* (Linnaeus, 1758)

*Culicoides punctatus* (Meigen, 1804)

*Culicoides riethi* Kieffer, 1914

*Culicoides riouxi* Callot & Kremer, 1961

*Culicoides salinarius* Kieffer, 1914

*Culicoides scoticus* Downes & Kettle, 1952

*Culicoides segnis* Campbell & Pelham-Clinton 1960

*Culicoides sphagnumensis* Williams, 1955

= *carjalaensis* Glukhova, 1957

*Culicoides stigma* (Meigen, 1818)

*Culicoides subfasciipennis* Kieffer, 1919

tribe Ceratopogonini Newman, 1834

***ALLUAUDOMYIA*** Kieffer, 1913

*Alluaudomyia needhami* Thomsen, 1935

= *pentaspila* Remm & Glukhova, 1971

*Alluaudomyia quadripunctata* (Goetghebuer, 1934)

***BRACHYPOGON*** Kieffer, 1899

**sg. *Isohelea*** Kieffer, 1917

*Brachypogon aquilonalis* (Clastrier, 1961)

*Brachypogon hyperboreus* (Clastrier 1961)

*Brachypogon incompletus* (Kieffer, 1925)

= *lapiae* Clastrier, 1961

*Brachypogon nitidulus* (Edwards, 1921)

= *finniae* Clastrier, 1961

*Brachypogon sociabilis* (Goetghebuer, 1920)

***CERATOPOGON*** Meigen, 1800

*Ceratopogon lacteipennis* Zetterstedt, 1838

*Ceratopogon niveipennis* Meigen, 1818

= *candidatus* Winnertz, 1852

***SCHIZOHELEA*** Kieffer, 1917

*Schizohelea leucopeza* (Meigen, 1804)

= *copiosa* Winnertz, 1852

= *xanthopeza* (Clastrier, 1963)

***SERROMYIA*** Meigen, 1818

*Serromyia atra* (Meigen, 1818)

*Serromyia femorata* (Meigen, 1804)

*Serromyia subinermis* Kieffer, 1919

tribe Heteromyiini Wirth, 1962

***CLINOHELEA*** Kieffer, 1917

*Clinohelea unimaculata* (Macquart, 1826)

= *variegata* (Winnertz, 1852)

tribe Sphaeromiini Newman 1834

***MALLOCHOHELEA*** Wirth, 1962

*Mallochohelea inermis* (Kieffer, 1909)

*Mallochohelea munda* (Loew, 1864)

= *dentata* (Kieffer, 1909)

*Mallochohelea nitida* (Macquart, 1826)

*Mallochohelea setigera* (Loew, 1864)

*Mallochohelea vernalis* Remm, 1965

***PROBEZZIA*** Kieffer, 1906

= ***Dicrobezzia*** Kieffer, 1919

*Probezzia concinna* (Meigen, 1818)

= venusta v. concinna (Meigen, 1818)

*Probezzia seminigra* (Panzer, 1798)

= *venusta* (Meigen, 1818)

= *borealis* (Clastrier, 1962)

***SPHAEROMIAS*** Curtis, 1829

*Sphaeromias fasciatus* (Meigen, 1804)

*Sphaeromias pictus* (Meigen, 1818)

= *candidatus* (Loew, 1856) preocc.

tribe Palpomyiini Enderlein, 1936

***BEZZIA*** Kieffer, 1899

*Bezzia annulipes* (Meigen, 1830)

*Bezzia bicolor* (Meigen, 1804)

*Bezzia circumdata* (Staeger, 1839)

= *solstitialis* (Winnertz, 1852)

*Bezzia coracina* (Zetterstedt, 1850)

= *albipes* (Winnertz, 1852)

*Bezzia fascispinosa* Clastrier, 1962

*Bezzia flavicornis* (Staeger, 1839)

= *flavipalpis* (Winnertz, 1852)

*Bezzia fuscifemoris* Remm, 1971

*Bezzia leucogaster* (Zetterstedt, 1850)

= *xanthocephala* Goetghebuer, 1911

*Bezzia nigritula* (Zetterstedt, 1838)

*Bezzia nobilis* (Winnertz, 1852)

= leucogaster var. punctata Lundström, 1910

*Bezzia winnertziana* Kieffer, 1919

= *gracilis* (Winnertz, 1852) nec Haliday, 1833

***PALPOMYIA*** Meigen, 1818

*Palpomyia armipes* (Meigen, 1838)

= *rufipecta* (Winnertz, 1852)

*Palpomyia distincta* (Haliday, 1833)

*Palpomyia flavipes* (Meigen, 1804)

= *hortulana* Meigen, 1818

*Palpomyia flavitarsis* (Meigen, 1838)

*Palpomyia lineata* (Meigen, 1804)

= *stagnalis* Clastrier, 1962

*Palpomyia lundstroemi* Remm, 1981

= *bispinosa* Lundström, 1916, preocc.

*Palpomyia serripes* (Meigen, 1818)

= *transfuga* (Staeger, 1839)

*Palpomyia tibialis* (Meigen, 1818)

## Notes

***Atrichopogon meloesugans* Kieffer, 1922**. This species was restored from synonymy with *Atrichopogon winnertzi* Goetghebuer, 1922 by Szadziewski et al. (2007).

***Dasyhelea modesta* (Winnertz, 1852)**. Old records reported before 1988 of this species (as *Dasyhelea aestiva* (Winnertz, 1852) or *Dasyhelea longipalpis* (Winnertz, 1852)) are questionable according to Dominiak and Szadziewski (2010). Records by Hämäläinen (1988) are valid.

***Ceratopogon neglectus* Winnertz, 1852**. Reported by Lundström (1910) as *Culicoides neglectus* from Finland. Szadziewski (1984) transferred it to *Dasyhelea*. Remm (1988) considered it a doubtful Ceratopogonidae. Because the type specimen is lost and the original description is insufficient it is considered nomen dubium by Dominiak and Szadziewski (2010). The species is removed from the Finnish list.

***Culicoides vexans* (Staeger, 1839)**. Reported from Finland by Lundström (1910) who listed ***Culicoides albicans*** as a probable synonym. *Culicoides vexans* was omitted in Frey and Storå (1941) and Havelka (1978) but included in Hackman (1980) and Szadziewski et al. (1997) (citing Hackman). According to Delecolle et al. (1983) Lundström’s *Culicoides vexans* represents *Culicoides albicans*. *Culicoides albicans* is included in the Finnish list and *Culicoides vexans* is removed from the Finnish fauna.

***Culicoides reconditus* (Campbell & Pelham-Clinton, 1960)** and ***Culicoides riouxi* Callot & Kremer, 1961**. *Culicoides riouxi* was treated as a synonym of *Culicoides reconditus* in Borkent and Wirth (1997) but restored from synonymy in Borkent (2012). Delecolle et al. (1983) reported *Culicoides riouxi* as a valid species from Finland (N: Tvärminne). The record of *Culicoides reconditus* by Hämäläinen (1988) from Finland (Ta: Riihimäki) was checked by Havelka but because he considered *Culicoides riouxi* and *Culicoides reconditus* synonyms the identity of Hämäläinen’s record remains unclear (see Havelka and Aquilar 1999 and Huldén et al. 2008 in accordance with Borkent and Wirth 1997). As a consequence *Culicoides reconditus* is not included in the Finnish list.

***Culicoides salinarius* Kieffer, 1914**. According to Delecolle et al. (1983) this species is indistinguishable from *Culicoides manchuriensis* Tokunaga, 1941 which is reported from Russian Karelia by Glukhova (1957).

***Probezzia concinna* (Meigen, 1818)**. The record listed by Krogerus (1960) from Kuusamo district (by mistake as ‘*coracina*’) is tentatively interpreted to be in the same locality in Finland as *Probezzia seminigra*, Kuusamo: Isosuo (Storå 1937). *Probezzia concinna* was listed as a variety of *Probezzia seminigra* in Frey and Storå (1941). Storå obviously identified both taxa. The species is here interpreted as a Finnish species.

***Palpomyia flavipennis* Meigen, 1804**. Misspelled species name for *Palpomyia flavipes* (Frey and Storå 1941).

***Palpomyia aterrima* Goetghebuer, 1921**. The record listed by Krogerus (1960) from Kuusamo district is from the Russian part according to locality data in Storå (1939). Not recorded in Finland.

***Palpomyia spinipes* (Meigen) in Panzer, 1806**. The record listed by Krogerus (1960) from Kuusamo district is from the Russian part according to the bog type information. Not recorded in Finland.
